# A Simple and Efficient Semen Cryopreservation Method to Increase the Genetic Variability of Endangered Mediterranean Brown Trout Inhabiting Molise Rivers

**DOI:** 10.3390/ani10030403

**Published:** 2020-02-29

**Authors:** Giusy Rusco, Michele Di Iorio, Roberta Iampietro, Stefano Esposito, Pier Paolo Gibertoni, Maurizio Penserini, Alessandra Roncarati, Nicolaia Iaffaldano

**Affiliations:** 1 Department of Agricultural, Environmental and Food Sciences, University of Molise, 86100 Campobasso CB, Italy; giusy.rusco@unimol.it (G.R.); michele.diiorio@unimol.it (M.D.I.); robertaiampietro@hotmail.it (R.I.); 2 Mediterranean Trout Research Group “MTRG”, 42037 Collagna RE, Italy; dott.stefanoesposito@gmail.com (S.E.); gibertoni@igiardinidellacqua.com (P.P.G.); mauriziopenserini.bio@gmail.com (M.P.); 3 School of Biosciences and Veterinary Medicine, University of Camerino, 62032 Camerino MC, Italy; alessandra.roncarati@unicam.it

**Keywords:** mediterranean brown trout, freezing procedure, sperm-to-egg ratio, conservation biology, sperm cryobank

## Abstract

**Simple Summary:**

The sperm cryobank is an effective strategy for protecting the biodiversity of the local brown trout population. Thus, the aim of the present work was to test, for the first time, the effectiveness of a semen cryopreservation protocol developed for cultivated salmonid fish on wild trout inhabiting the Molise rivers. Moreover, the effects of two different thawing rates (40°C/5 s and 10°C/30 s) were evaluated in vitro and in vivo. In addition, different sperm-to-egg ratios (6 × 10^5^:1, 4.5 ×10^5^:1 and 3 × 10^5^:1) were also investigated in vivo in order to find an effective freezing protocol useful for the creation of the first European semen cryobank for the Mediterranean brown trout (*S. macrostigma = S. cettii*). From results obtained in vitro 40 °C turned out to be the best thawing rate, which in combination with a sperm-to-egg ratio of 4.5 × 10^5^:1 was, in vivo, the highest fertilization rate. The important results obtained in the present work corroborated the remarkable validity and effectiveness of the freezing protocol used in this study on the native trout populations from Molise.

**Abstract:**

The aim of our study was to test the effectiveness of a simple semen cryopreservation procedure, developed for cultivated salmonid, on the wild salmonid of the Mediterranean area and to evaluate the effect of different thawing rates and sperm-to-egg ratios. The semen of five individual males was diluted into a final extender concentration of 0.15 M glucose and 7.5% methanol and loaded into 0.25 mL plastic straws, and a final sperm concentration of 3.0 × 10^9^ sperm/mL was obtained. After equilibration, the straws were frozen by exposure to liquid nitrogen vapor at 3 cm above the liquid nitrogen level for 5 min. The semen was thawed at 40 °C/5 s or 10 °C/30 s. The sperm cryosurvival was evaluated by examining in vitro the sperm motility parameters using the CASA system, followed by fertilization trials in vivo, using three different sperm-to-egg ratios 6 × 10^5^, 4.5 × 10^5^ and 3 × 10^5^:1. The applied cryopreservation procedure resulted in remarkably high (85.6%) post-thaw sperm total motility, when the semen was thawed at 40 °C/5 s, whilst the highest fertilization rate (53.1%) was recorded for a sperm-to-egg ratio of 4.5 × 10^5^:1. According to these outcomes, the cryopreservation procedure that was tested turned out to be effective for the wild population of Mediterranean brown trout and practical for the creation of the first European semen cryobank foreseen as part of our “LIFE” Nat.Sal.Mo. project.

## 1. Introduction 

Mediterranean brown trout is currently listed in Annex II of the Habitat Directive (under the taxon *Salmo macrostigma*) and its overall conservation status declared at the EU level as "bad" and “in decline”. In such a contest, the situation of the Italian populations significantly contributes to the overall EU status because the Mediterranean trout populations largely represent the majority of the European populations of this species. It is currently listed in the Italian IUCN Red List as critically endangered [1] under the accepted taxon for the Italian population, *S. cettii*. Over the past decades, the decline of these native populations was mainly attributed to habitat loss, poorly regulated fishing and genetic introgression following the introduction of non-native strains. The genetic introgression of native populations caused a loss of their ecological specialization characteristics, such as adaptation to global changes, resistance to diseases, etc.

In this regard, the project “LIFE” Nat.Sal.Mo, recently funded by the EU, aims to ensure the recovery and the conservation of native Mediterranean trout (*S. macrostigma = S. cettii*) in Molise river basins (Molise region—Southern Italy). 

Among the specific objectives of the project an important role is the restoration of genetic integrity. In this matter, we foresee to reduce the genetic introgression in the native populations of the project area using two main strategies: (1) to allow access to the main natural spawning grounds only to non-introgressed wild breeders; and (2) to use a part of the pure wild breeders for artificial reproduction with frozen semen combined with appropriate fertilization schemes in order to increase the genetic variability of the offspring. In particular, this last action includes frozen sperm doses from a large number of native breeders for the creation of the first semen cryobank as an effective strategy for protecting the biodiversity of the local Mediterranean brown trout populations. 

However, a fundamental assumption for the creation of a sperm cryobank is the development of a successful freezing protocol. Indeed, it is known that cryopreservation procedures are highly stressful for sperm cell [2]. Cell damage can be produced both within the cells due to osmotic or oxidative stress and in the external medium by mechanical forces due to ice crystal formation [3]. 

Moreover, it is to be highlighted that the semen from salmonids is among the most difficult to cryopreserve due to its peculiar features (short duration of motility, low adenosine triphosphate production, high sensitivity to osmotic stress and high number of spermatozoa required to fertilize one egg), which make them more sensitive to the cryopreservation process [4]. Thus, the conservation of cell structure and its functionality strictly depends on the cryopreservation protocol used. In this regard, in the past years our research group has been perfecting an effective semen cryopreservation protocol for the Mediterranean trout of the Molise rivers, with the aim of reducing cell damage and preserving the fertilizing ability of sperm [5,6,7]. However, despite our efforts and promising results obtained so far, we still aim to obtain a freezing procedure reporting a fertilization rate as similar as possible to that of fresh semen. Recently, a simple and efficient semen cryopreservation method for several cultivated salmonid species was developed by some Polish researchers, which attracted our interest [8,9,10,11]. These authors suggested that this freezing method could be “universal” for the semen cryopreservation of salmonids species, attributing its effectiveness to the composition of the freezing medium (glucose–methanol extender). 

Thus, the aim of the present work was to test, for the first time, the effectiveness of the aforementioned semen cryopreservation protocol on wild trout inhabiting the Molise rivers. Moreover, the effects of two different thawing rates (40 °C/5 s and 10 °C/30 s) were evaluated in vitro and in vivo and different sperm-to-egg ratios (6 × 10^5^:1, 4.5 × 10^5^:1 and 3 × 10^5^:1) were also investigated on fertilization rate. The obtaining of an effective freezing protocol represents a milestone in the LIFE Nat.Sal.Mo project useful for the creation of the first European semen cryobank for the Mediterranean brown trout (*S. macrostigma*).

## 2. Materials and Methods

### 2.1. Collection of Sperm and Eggs

Sperm and eggs were obtained from Mediterranean brown trout caught from the Biferno river (Molise region, latitude: 41°28′47.8″ N and longitude: 14°28′40.9′ E) during spawning season (January-February 2019) by electro-fishing.

Sperm samples were collected by the gentle abdominal massaging of five individual males; abdomens and urogenital papilla were dried before stripping, with special care to avoid contamination of semen with urine, mucus and blood cells. Following samples collection in the river, the tubes containing sperm were transferred to the laboratory in a portable refrigerator at 4 °C within 30 min. Only spermatozoa that showed a motility rate higher than 70% were used in the experimental design.

Eggs were collected from two females stripped by gentle abdominal massage in a dry metal bowl and were checked visually to ensure that those used in the fertilization experiments were well-rounded and transparent. The fish were aged as 2 to 3 + years, and the average total lengths of the fishes were 25.4 ± 5.2 cm for males and 30.5 ± 6.7 cm for females.

The experiments were conducted in accordance with the Code of Ethics of the EU Directive 2010/63/EU for animal experiments. This study is part of a Nat.Sal.Mo LIFE project that received “a positive opinion” from the Ministry of the Environment and the Protection of the Territory and the Sea. 

### 2.2. Sperm Cryopreservation

The semen cryopreservation protocol based on the above mentioned procedure [10], which uses a simple glucose–methanol (GM) extender, was used. Briefly, the semen of five individual males was diluted to a final extender concentration of 0.15 M glucose and 7.5% methanol and loaded into 0.25 mL plastic straws, obtaining a final sperm concentration of 3.0 × 10^9^ sperm/mL. Subsequently, the straws were placed on a 3 cm-high frame and equilibrated for 15 min on ice. After equilibration, the straws were frozen by exposure to liquid nitrogen vapor at 3 cm above the liquid nitrogen level for 5 min. They were then placed in liquid nitrogen. The straws were then thawed by immersion in a water bath at two different thawing rates: at 40 °C for 5 s, as reported in the original protocol by Nynca et al. [10], or at 10 °C for 30 s, as already used in our previous work [7].

### 2.3. Sperm Analysis

The sperm motility was evaluated in both fresh and thawed semen. For frozen semen, the analyses were carried out in duplicate (two straws for each treatment). Moreover, the fresh semen concentration was measured by the Neubauer chamber. Briefly, the semen was extended 1/1000 (v:v) with 3% NaCl (w:v), and sperm counts were carried out in duplicate, at a magnification of 400× and expressed as × 10^9^/mL. 

The sperm motility parameters were examined using a Computer-Assisted Sperm Analysis (CASA) system coupled to a phase contrast microscope (Nikon model Ci-L) employing the Sperm Class Analyzer (SCA) software (VET Edition, Barcelona, Spain). Spermatozoa were activated at a dilution ratio of 1:300 in a solution consisting of 1 mM CaCl_2_, 20 mM Tris, 30 mM glycine and 125 mM NaCl, at pH 9.0 and supplemented with 0.5% bovine albumin [12]. The following sperm motility parameters were evaluated: motile spermatozoa (MOT, (%)), curvilinear velocity (VCL, (μm/s)), straight-line velocity (VSL, (μm/s)), average path velocity (VAP, (μm/s)), linearity (LIN, (%)) and straightness (STR, (%)).

### 2.4. In Vivo Reproductive Ability of Cryopreserved Semen 

**Figure 1:**
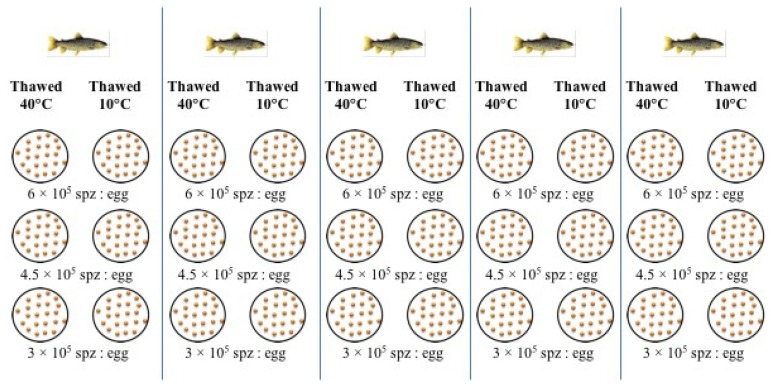
Fertilization scheme used to test the spermatozoa fertilization ability of five individual males thawed at 40 °C or 10 °C with sperm-to-egg ratios of 600,000:1, 450,000:1 and 300,000:1.

The fertilization trial was performed on March 5, 2019. Pooled eggs from two females were divided into batches of 106 ± 8 eggs, using 34 glass laboratory jars: four jars were fertilized using excess fresh semen at the beginning and at the end of the fertilization trial (control groups) in order to test the quality of the eggs; and 30 jars were divided into six treatment groups. Each treatment group was fertilized using semen of the individual males (*n* = 5) thawed at 40 °C or 10 °C with three different spermatozoa-to-egg ratios (6 × 10^5^:1, 4.5 × 10^5^:1 and 3 × 10^5^:1), as shown in Figure 1. Before adding the semen, 5 mL of fertilization solution D532 (20mM Tris, 30 mM glycine, 125 mM NaCl, pH 9.0) [12] was added to the eggs. The sperm was gently mixed with the eggs for 10 s, and then about 20 mL of hatchery water was added. After 2 min, the eggs were washed with hatchery water and placed in an incubator with running water at about 10 °C, where they developed until the eye stage (after ~25–30 days from fertilization). Unfertilized and dead eggs were counted and removed twice a week. The fertilization success was established by calculating the percentage of embryos at the eyed stage, using the initial number of eggs (number of eyed eggs × initial egg number^-1^ × 100).

### 2.5. Statistical Analysis 

Sperm motility parameters and fertilization rate measured across the different treatments were compared by analysis of variance (ANOVA) followed by Duncan’s comparison test. To assess the fixed effects of thawing rate, sperm/egg ratio and their interaction in vivo, we used the generalized linear model (GLM) procedure. Significance was set at *p* < 0.05. All statistical tests were conducted using the software package SPSS (SPSS 15.0 for Windows, 2006; SPSS, Chicago, IL, USA). 

## 3. Results

### 3.1. In Vitro Sperm Quality of Fresh and Cryopreserved Semen 

Fresh semen was characterized by a high percentage of sperm motility (92.7% ± 1.0 %). The average sperm concentration was 15.7 ± 3.2 × 10^9^ sperm/mL. 

Total motility decreased significantly after semen cryopreservation for both thawing rates tested (Table 1). However, a significant (*p* < 0.05) effect of thawing rate was recorded. In particular, the high thawing rate (40 °C/5 s) resulted in a higher percentage of motile sperm (85.6% ± 2.2%) in comparison to sperm total motility obtained in semen thawed at 10 °C/30 s (79.8% ± 1.2%).

The cryopreservation procedure did not significantly influence the following motility kinetic parameters: VCL, VAP, STR and LIN; the exception thus was for VSL, whose value increased significantly when the semen was thawed at 40 °C/5 s.

### 3.2. In Vivo Fertilizing Ability of Fresh and Cryopreserved Semen 

The percentage of eyed embryos for fresh semen was significantly higher (85.1% ± 1.4%) compared to frozen semen (Table 2). A significant effect of the thawing rate on the fertilization ability of the cryopreserved semen was observed (*p* < 0.05), whilst no significant effect of the sperm-to-egg ratio and interaction effect between these two variables were observed.

The highest percentage of eyed embryos was found when the semen were thawed at 40 °C, using a sperm-to-egg ratio of 4.5 × 10^5^ (53.2% ± 6.9%), although significant differences were observed only with samples thawed at 10°C and at the sperm-to-egg ratios of 4.5 × 10^5^:1 and 3 × 10^5^:1. 

## 4. Discussion

An effective semen cryopreservation method was recently developed by a Polish research group for several cultivated salmonid species [8,9,10,11,13]. The high post-thaw semen quality (total motility above 85%) reported in brown trout (*Salmo trutta* m. *fario*), rainbow trout (*Oncorhynchus mykiss*), sea trout (*Salmo trutta* m. *trutta*), brook trout (*Salvelinus fontinalis*), Atlantic salmon (*Salmo salar)* and whitefish, as well as the remarkable fertilizing ability (eyed embryos and hatching rates above 80%) recorded in brown trout, attracted our interest, and prompted us to test the effectiveness of this semen freezing protocol on the wild salmonid of the Mediterranean area (*S. macrostigma* = *S. cettii*). Moreover, two different thawing rates (40 °C for 5 s and 10°C for 30 s) and different sperm-to-egg ratios (6 × 10^5^:1, 4.5 × 10^5^:1 and 3 × 10^5^:1) were also evaluated in order to standardize the cryopreservation protocol for our native trout. 

The freezing procedure tested here turned out to have a positive impact on sperm total motility post-thawing, recording values above 80%. These results were remarkably better compared to that found in our previous studies, which ranged between 33% and 52% [5,6,7]. However, a significant decrease in the total motility after thawing compared to the fresh semen was recorded, although this is expected following the cryopreservation process. In fact, it is known that the freezing and thawing of sperm is a complex process that causes several forms of cellular damages [2,5]. These injuries have been mainly attributed to cold shock, extreme osmotic changes, intracellular ice crystals and reactive oxygen species, with the consequent reduction of the membrane’s permeability and integrity, motility, viability and fertilizing ability [14]. One of main critical factors that affects the cryosurvival of spermatozoa is the formation of intracellular ice crystals (ice injury) [2,5]. Therefore, the choice of the optimal freezing and thawing rate is a fundamental requirement in preventing intracellular ice recrystallization and irreversible sperm membrane damage. 

In particular, we recorded higher post thawing total motility values (above 80%) using the thawing rate of 40 °C/5 s with respect to 10 °C/30 s. Thus, a higher warming temperature (40 °C) over a shorter period (5 s) was better than a slightly longer exposure to a lower temperature (10 °C for 30 s). These results are also confirmed in vivo; in fact, a significant (*p* < 0.05) effect of the thawing rate was observed on fertilization ability. Other authors also found that a higher thawing temperature ensured better sperm cryosurvival [9,13,15,16]. On the contrary, in our previous research, the thawing temperature of 10 °C registered both a higher post-thaw semen quality and fertilization ability with respect to those recorded at 30°C/10 s [7]. These conflicting results could be attributed to the different experimental conditions adopted in the two studies (extender composition, freezing rates and equilibration time). However, the rationale to test the thawing temperature of 10 °C in both studies was driven from the possibility to use the spring water (temperature ranging from 7 to 12 °C) to thaw the straws, thus facilitating the on-field artificial reproduction practices.

In discordance with the total motility parameter after thawing, we found an unexpected increase in the kinetic motility parameters with respect to those of fresh semen, although this is not statistically significant. Between the thawing temperature tested the differences were also not significant, except for VSL, which was significantly higher both in respect to fresh semen and in semen thawed at 10 °C. Further studies are needed to confirm these results and eventually to explain what the reasons are for the increase of the kinetic motility parameters in the post thawing semen. Nynca et al. [13], in a recent study on semen of cultivated brown trout, found a similar trend; in fact, a significant increase in all kinetic motility parameters in response to freezing semen was recorded, excepted for VCL.

Another crucial point considered in this work was the identification of the optimal sperm/egg ratio. No significant effect for the sperm/egg ratio was found, although 4.5 × 10^5^:1 registered the best eyed embryos percentage compared to the other ones tested (6 × 10^5^:1 and 3 × 10^5^:1). Nynca et al. [9] showed the highest percentage of eyed embryos for both the 3 × 10^5^:1 and 6 × 10^5^:1 sperm-to-egg ratios tested. In accordance with the same authors, a controlled and low sperm-to-egg ratio improves the effectiveness of brown trout cryopreservation and its fertilization ability. The optimal sperm/egg ratio discovered in this work allows the reduction of the concentration of spermatozoa necessary for egg fertilization, without decreasing the hatching rates. In this way it is possible to limit the wastage of sperm [17], which would be valuable for this endangered species.

Although, we did not obtain the same excellent results that were reported our Polish colleagues who recorded a fertilization rate close to that of fresh semen, we think that the freezing semen protocol standardized here resulted as effective even for the autochthonous trout populations from Molise. It is a practical freezing protocol that includes the use of a simple glucose–methanol extender and a reduced sperm-to-egg ratio by a 10 factor, resulting as better with respect to our previous freezing protocol [7] whilst maintaining similar fertilization rates (5 × 10^6^:1 sperm/egg ratio; 54.5% fertilization rate vs. 4.5 × 10^5^:1 sperm/egg ratio; 53.2% fertilization rate). 

The sampled individuals in our study were not cultivated brown trout but wild breeders of a Mediterranean trout with wide variability of biological features, leading to high heterogeneity both in semen quality traits and reproductive performance among individuals within the same population [5,18]. These biological and physiological differences can affect the freezability of individual sperm, causing inter-male variability in the responses to cryopreservation. In this regard, to ensure genetic variability in supportive breeding of wild native populations, it is important to use sperm coming from a single male in order to avoid possible sperm competition in semen pools [19]. Regarding the LIFE Nat.Sal.Mo. project, the creation of a sperm cryobank from genotyped individuals allows us to store sperm doses obtained from a large contingent of wild males, which will always be available, for cross-fertilization schemes [20]. Thus, the use of cryopreserved semen in artificial fertilization protocols represents a valuable tool in maintaining the genetic diversity and the fitness within self-sustaining populations.

## 5. Conclusions

Summing up, the important results obtained in the present work corroborated the validity and effectiveness of the freezing protocol used in this study even for the autochthonous trout populations from Molise (*S. macrostigma = S. cettii*). 

The high post-thaw semen quality and the reduced concentration of spermatozoa necessary for egg fertilization represented an important goal to be achieved in order to contribute to the creation of the first European sperm cryobank aiming at the restoration of Mediterranean brown trout in Molise (Italy), a milestone of our European project "LIFE Nat.Sal.Mo". In addition, further research is planned in order to understand the biological mechanisms involved in the variability among the breeders. 

## Figures and Tables

**Table 1:** Computer-Assisted Sperm Analysis (CASA) parameters recorded for fresh semen or frozen semen (mean ± SE) undergoing two different thawing rates (*n* = 5).

Treatment	Total motility (%)	VCL (μm/s)	VAP (μm/s)	VSL (μm/s)	STR (%)	LIN (%)
Fresh	92.7 ± 1.0 ^a^	41.3 ± 5.0 ^a^	29.3 ± 3.8 ^a^	17.3 ± 1.7 ^b^	64.4 ± 2.6 ^a^	49.2 ± 4.3 ^a^
Thawed 40 °C	85.6 ± 2.2 ^b^	57.8 ± 8.4 ^a^	52.7 ± 7.4 ^a^	34.7 ± 2.3 ^a^	73.2 ± 5.0 ^a^	67.2 ± 5.3 ^a^
Thawed 10 °C	79.8 ± 1.2 ^c^	52.7 ± 14.1 ^a^	44.3 ± 11.7 ^a^	24.2 ± 3.1 ^b^	62.9 ± 7.7 ^a^	53.9 ± 8.3 ^a^

^a,b,c^ Values within a column reporting different superscript letter differ significantly at *p* < 0.05. VCL: curvilinear velocity; VAP: average path velocity; VSL: straight-line velocity; STR: straightness; LIN: linearity.

**Table 2:** Fertilization ability of fresh or frozen semen (mean ± SE) undergoing two thawing rates and using different sperm-to-egg ratios (*n* = 5).

Semen Treatment	Thawing Rate (°C)	Sperm/egg Ratio	Fertilization Rate (%)
Fresh			85.1 ± 1.4 ^a^
Frozen	40	600,000:1	46.1 ± 7.3 ^bc^
		450,000:1	53.2 ± 6.9 ^b^
		300,000:1	38.9 ± 10.5 ^bc^
	10	600,000:1	41.0 ± 5.9 ^bc^
		450,000:1	30.8 ± 3.8 ^c^
		300,000:1	27.3 ± 5.2 ^c^
Thawing effect Sperm/egg ratio effect Thawing rate × sperm/egg ratio effect			*p* = 0.031 *p* = 0.287 *p* = 0.464

^a,b,c^ Different superscript letters within the same column indicate a significant difference (*p* < 0.05).

## References

[1] Bianco P.G., Caputo V., Ferrito V., Lorenzoni M., Nonnis Marzano F., Stefani F., Sabatini A., Tancioni L., Rondinini C., Battistoni A., Peronace V., Teofili C. (2013). Pesci d’acqua dolce. Lista Rossa IUCN dei Vertebrati Italiani.

[2] Cabrita E., Sarasquete C., Martínez-Páramo S., Robles V., Beirao J., Pérez-Cerezales S., Herráez M.P. (2010). Cryopreservation of fish sperm: Applications and perspectives. J. Appl. Ichthyol..

[3] Watson P.F. (2000). The causes of reduced fertility with cryopreserved semen. Anim. Reprod. Sci..

[4] Martínez-Páramo S., Pérez-Cerezales S., Gòmez-Romano F., Blanco G., Sánchez J.A., Herráez M.P. (2009). Cryobanking as tool for conservation of biodiversity: Effect of brown trout sperm cryopreservation on the male genetic potential. Theriogenology.

[5] Iaffaldano N., Di Iorio M., Manchisi A., Esposito S., Gibertoni P.P. (2016). Effective freezing rate for semen cryopreservation in endangered Mediterranean brown trout (*Salmo trutta macrostigma*) inhabiting the Biferno river (South Italy). Zygote.

[6] Di Iorio M., Esposito S., Rusco G., Roncarati A., Miranda M., Gibertoni P.P., Cerolini S., Iaffaldano N. (2019). Semen cryopreservation for the Mediterranean brown trout of the Biferno River (Molise-Italy): Comparative study on the effects of basic extenders and cryoprotectants. Sci. Rep..

[7] Rusco G., Di Iorio M., Gibertoni P.P., Esposito S., Penserini M., Roncarati A., Cerolini S., Iaffaldano N. (2019). Optimization of Sperm Cryopreservation Protocol for Mediterranean Brown Trout: A Comparative Study of Non-Permeating Cryoprotectants and Thawing Rates In Vitro and In Vivo. Animals.

[8] Ciereszko A., Dietrich G.J., Nynca J., Dobosz S., Zalewski T. (2014). Cryopreservation of rainbow trout semen using a glucose–methanol extender. Aquaculture.

[9] Nynca J., Dietrich G.J., Dobosz S., Grudniweska J., Ciereszko A. (2014). Effect of cryopreservation on sperm motility parameters and fertilizing ability of brown trout semen. Aquaculture.

[10] Nynca J., Judycka S., Liszewska E., Dobosz S., Ciereszko A. (2017). Standardization of spermatozoa concentration for cryopreservation of rainbow trout semen using a glucose-methanol extender. Aquaculture.

[11] Judycka S., Nynca J., Liszewska E., Dobosz S., Grudniewska J., Ciereszko A. (2018). Optimal sperm concentration in straws and final glucose concentration in extender are crucial for improving the cryopreservation protocol of salmonid spermatozoa. Aquaculture.

[12] Billard R. (1992). Reproduction in rainbow trout: Sex differentiation, dynamics of gametogenesis, biology and preservation of gametes. Aquaculture.

[13] Nynca J., Judycka S., Liszewska E., Dobosz S., Grudniewska J., Arai K., Fujimoto T., Ciereszko A. (2016). Utility of different sugar extenders for cryopreservation and post-thaw storage of sperm from Salmonidae species. Aquaculture.

[14] Moghanloo K., Niksirat H., Mojazi Amiri B., Mirtorabi S.M. (2007). The effects of extender type, freezing and thawing rates on fertility of the cryopreserved semen of the Caspian brown trout (*Salmo trutta caspius*). Iran. J. Fish. Sci..

[15] Fowler A., Toner M. (2006). Cryo-injury and biopreservation. Ann. Ny Acad. Sci..

[16] Sarvi K., Niksirat H., Amiri B.M., Mirtorabi S.M., Rafiee G.R., Bakhtiyari M. (2006). Cryopreservation of semen from the endangered Caspian brown trout (*Salmo trutta caspius*). Aquaculture.

[17] Mongkonpunya K., Pupipat T., Tiersch T.R. (2000). Cryopreservation of sperm of Asian catfishes, including the endangered Mekong giant catfish. Cryopreservation in Aquatic Species.

[18] Iaffaldano N., Di Iorio M., Manchisi A., Gibertoni P.P., Esposito S. (2016). Semen quality of threatened native population of Mediterranean brown trout (*Salmo cettii*, Rafinesque 1810) in the Biferno River (Molise Region-South Italy). Turk. J. Fish. Aquat. Sc..

[19] Beirão J., Egeland T.B., Purchase C.F., Nordeide J.T. (2019). Fish sperm competition in hatcheries and between wild and hatchery origin fish in nature. Theriogenology.

[20] Dupont-Nivet M., Vandeputte M., Haffray P., Chevassus B. (2006). Effect of different mating designs on inbreeding, genetic variance and response to selection when applying individual selection in fish breeding programs. Aquaculture.

